# Plasticity of Respiratory Function Accommodates High Oxygen Demand in Breeding Sea Cucumbers

**DOI:** 10.3389/fphys.2020.00283

**Published:** 2020-04-02

**Authors:** Xiaoshang Ru, Libin Zhang, Shilin Liu, Hongsheng Yang

**Affiliations:** ^1^CAS Key Laboratory of Marine Ecology and Environmental Sciences, Institute of Oceanology, Chinese Academy of Sciences, Qingdao, China; ^2^Laboratory for Marine Ecology and Environmental Science, Qingdao National Laboratory for Marine Science and Technology, Qingdao, China; ^3^Center for Ocean Mega-Sciences, Chinese Academy of Sciences, Qingdao, China

**Keywords:** marine invertebrate, respiratory function, oxygen demand, reproduction, physiological mechanism, oxidative stress

## Abstract

Physiological plasticity allows animals to adjust their physiological function to abiotic and biotic variations. It has been mostly studied in the context of response to external factors and not much is known on how animals adjust their physiology to cope with variations in internal conditions. The process of reproduction implies gonadal maturation and other internal changes, bringing various challenges to the animal such as an increased demand for energy and oxygen. Here, the capacity of the sea cucumber, *Apostichopus japonicus* to adjust its respiratory function and physiological mechanisms during reproduction was studied using a time-lapse videography and metabolomics approach. The results showed that reproduction caused a significant increase in oxygen consumption in *A. japonicus*. Interestingly, breeding sea cucumbers can accommodate the high oxygen demand by accelerating respiratory rate. However, to maintain a necessary high level of respiratory activity during reproduction, sea cucumbers need consume large amounts of adenosine triphosphate (ATP). In addition, the metabolomic data suggests that oxidative stress and hormone regulation are the physiological mechanisms linking reproduction and respiratory function. Altogether, these findings suggest that plasticity of respiratory function is an effective tactic to cope with high oxygen demand during reproduction.

## Introduction

Reproduction poses challenges because gametes, also other behavioral and physiological functions associated with reproduction, require large amounts of energy, nutrients, and oxygen (Speakman, 2008; Lopes et al., 2010; McBride et al., 2015; Nie et al., 2015). As a result, these large demands may aggravate the physiological burden and impact the functioning of the digestive system, motor functions, respiratory and immune systems, and even impair survival of the gravid animal (Weeks, 1996; Alonso-Alvarez et al., 2004; Harshman and Zera, 2007; Lu et al., 2015). Notably, of these demands, oxygen is needed for efficient metabolic activity (Wenger, 2000; Nelson, 2016). Previous studies have reported that high oxygen consumption, resulting from increases basal metabolic rate, exists extensively in pregnant mammals, as well as gravid birds, reptiles, insects, fish, crustaceans, and shellfish throughout the whole breeding season (Sláma, 1964; Nilsson and Råberg, 2001; Wilhelm Filho et al., 2001; Baeza and Fernández, 2002; Mao et al., 2006; Sparling et al., 2006; Foucart et al., 2014). Such changes in metabolism may have a strong correlation with courtship behavior as well as with embryo development and parental care.

In response to variations in abiotic (i.e., decreasing ambient oxygen concentration) and biotic (i.e., increasing competition for food) challenges, animals have evolved the capacity to adjust behavior and physiology to environmental conditions. Plasticity in respiratory physiology, associated with variation in energy demands, has been mostly addressed in the context of responses to external factors (e.g., stress and temperature) and not much is known on how animals are able to adjust this function to variation in internal conditions (Barker et al., 2011; Zhao et al., 2011), in particular those associated with reproduction. Some examples of plasticity in respiratory function include: (i) increased respiratory rate to acquire more oxygen (Williams and Tieleman, 2000; Claireaux and Chabot, 2016); (ii) increased oxygen binding capacity of hemoglobin or heart rate to improve oxygen transport efficiency (Birchard et al., 1984; Wu, 2002); and (iii) change in oxygen allocation patterns (Plaut, 2002). These possible responses suggest that the plasticity of physiological functions is crucial for the homeostasis of oxygen balance in animals.

Respiratory organs such as lungs and gills serve to procure steady-state levels of oxygen to the organism. Previous studies have shown that respiratory organs of gravid animals will show functional adaptations to meet the increasing oxygen demands of reproduction (Speakman and McQueenie, 1996). Respiratory organs work intensively over breeding periods to ensure successful reproduction. Some of the physiological changes in respiratory organs during these hypermetabolic periods involve increasing aerobic metabolism in mitochondria, accelerating consumption of energy reserves, and elevating secretion of metabolic hormones (Sláma, 1964; Morgan et al., 1997). However, despite research indicating these physiological changes, little is known about the precise molecular relationship between the plasticity of respiratory function and underlying regulatory mechanisms during reproduction.

In the present study, we explored the hypothesis that the plasticity of respiratory functions in accommodating high oxygen demands in a marine invertebrate during reproduction. The sea cucumber *Apostichopus japonicus*, is an iteroparous echinoderm found in the northwest Pacific Ocean (Zhang et al., 2017). *A. japonicus* is an ideal model for physiological studies, because the physiological patterns of the adults are sensitive to environmental and internal physiological changes (Ji et al., 2008; Sun et al., 2018b). Previous studies have shown that investigating the respiratory movement of free-range aquatic animals is difficult (Heath, 1972). To address this problem, we have used a contactless time-lapse camera to record the respiratory behavior of experimental animals without additional stimulus. In addition, both ultra-performance liquid chromatography and quadrupole time-of-flight mass spectrometry (UPLC-Q-TOF-MS) were used to analyze the metabolic profiling of the animal (Macel et al., 2010).

Our objectives were: (i) to elucidate the relationship between reproduction, oxygen consumption and respiratory function in *A. japonicus*; and (ii) to explore the metabolic mechanisms underlying plasticity in respiratory behavior during reproduction. Therefore, our study provides new insights into how marine invertebrates, such as the sea cucumber, accommodate reproductive burden through plasticity in respiratory function.

## Materials and Methods

### Ethical Note

All procedures involved in animal collection, rearing and dissection were performed under the Guideline of Ethical Regulations of Animal Welfare of the Institute of Oceanology, Chinese Academy of Sciences (IOCAS 2013.3). Our research protocols were approved by the Animal Welfare Committee of the IOCAS.

### Study Species and Area

*Apostichopus japonicus* is a temperate species, with an annual reproductive cycle determined by ambient temperature (Wang et al., 2015). In December 2016, a total of 200 *A. japonicus* (160 ± 30 g) were collected from Rushan Bay (36°47′ N, 121°34′ E) at 8°C, Shandong Province, China. Animals were transferred to the laboratory and acclimated to 14°C, an optimal temperature for gonad growth (Liu et al., 2015), at a rate of 0.5°C per day for 2 weeks before the experiment.

### Experimental Design and Rearing Conditions

During the experiment, animals were held in four 6 m^3^ tanks at 14 ± 1°C using eight OKE-6710HF heating systems (Sewon Oke Co., Ltd., Seoul, South Korea). The animals were fed a specific diet for *A. japonicus* broodstock (Shandong Oriental Ocean Sci-Tech Co., Ltd., Yantai, China). To maintain good water quality, uneaten food and feces were siphoned from the tanks at 8:00 am daily and half of the used water was changed. Dissolved oxygen was ∼8.2 mg L^–1^, pH was ∼8.1, salinity was 31‰, the level of ammonia was less than 0.24 mg L^–1^, light intensity was 25 Lx and photoperiod was 12 h light:12 h dark.

The study lasted for 70 days. After acclimation to 14°C, 28 females were randomly selected for sampling at day 0 (non-breeding stage, NBS) and day 70 (breeding stage, BS). On each sampling day, 20 different adults were used to record respiratory behavior and oxygen consumption rate (OCR). Eight adults were used for metabolomic and adenosine triphosphate (ATP) content analysis.

### Respiratory Rate

The respiratory tree is a specialized organ for gas exchange and excretion in sea cucumbers, anatomically attached to the cloaca by connective tissue (Gao and Yang, 2015). Therefore, the respiratory rate can be determined by cloacal movement (Gianasi et al., 2015; Sun et al., 2018a).

For behavioral observations, each animal was placed in a 20 L transparent glass tank at the same temperature and water quality as described above. Over 30 min of acclimation under experimental conditions, the females distributed themselves freely in the tanks. A TLC 200 Brinno time-lapse camera (Brinno Co., Ltd., Taibei, China) equipped with a height-adjustable tripod (Tushihui Co., Ltd., Guangzhou, China) was placed outside the tank focused on one animal at a time to record the respiratory movements. During recording, the camera was set to take photos at 1 s intervals for 20 min automatically. The videos were stored as AVI files with a resolution ratio of 640 × 480 pixels and a frame rate of 10 fps. Videos were analyzed by the “slow-motion” function of the Coreplayer software (Baofeng Technology Ltd., Beijing, China). In details, the videos were played in slow forward mode at a one-tenth speed. Then the frequency of cloacal movements counted per minute served as the respiratory rate.

### Oxygen Consumption Rate

Oxygen consumption rate was measured in 10 NBS and 10 BS individuals and calculated according to an established method for *A. japonicus* (Dong et al., 2006; Bai et al., 2018; Huo et al., 2018). Feeding was not provided for 24 h before OCR measurement to allow for gut evacuation, while not causing starvation stress. Each animal was put into a 2.8 L sealed glass container (Subo Co., Ltd., Chongzhou, China), filled with seawater and without aeration. There were also twelve blank controls to correct for respiration of microorganisms in water. During OCR measurements, the temperature, salinity, and light density in the respiratory chamber were the same as in the tanks above. After 2 h, oxygen levels of each water sample was determined using the ECO Sense ODO200 Dissolved oxygen meter (YSI Co., Ltd., Yellow Springs, OH, United States), calibrated using the Winkler method (Huo et al., 2018).

### Sample Collection

Sea cucumbers were euthanized by decollation and the whole respiratory tree and gonadal tissues were taken out of the body cavity from the incision site. Only females were considered in our study. As female and male *A. japonicus* can not be differentiated by their external morphologies, we identified the females by visualizing oocytes in the gonad under a light microscope (Olympus Co., Ltd., Tokyo, Japan). During dissection, the total body, gutted body, gonad and respiratory tree of each animal were weighed using a LT1002B electronic balance.

The reproductive stage was determined by the gonad somatic index (GI) calculated as GI = 100×GW/BW, where GW and BW are the weight of gonad and gutted body wall, respectively (Liu et al., 2015; Wang et al., 2015). The respiratory tree was washed by ultrapure water, gently wiped by sterile gauze, and a 1 g sample was stored in a tube at −80°C for future analysis.

### UPLC-Q-TOF-MS Analysis

For metabolomics, 10 μl of L-2-chlorophenylalanine was added to each respiratory tree sample (50 mg) as an internal standard. Extraction proceeded with the addition of 800 μl of methanol, mixing and grounding to a fine powder with a grinding mill. After vortexing for 30 s, the sample was centrifuged at 13,400 *g* for 15 min at 4°C. An aliquot of the supernatant (200 μl) was transferred to a clean vial for subsequent analysis.

Chromatographic separation was performed via Agilent 1290 Infinity LC system (Agilent Technologies, Santa Clara, CA, Untied States) with an Agilent C18 column (Agilent Technologies) maintained at 40°C. Other parameters for chromatographic separation were as follows: the injection volume was 4 μl, the automatic injector temperature was 4°C, the flow rate was 0.35 ml min^–1^, and the mobile phase consisted of A (ultrapure water with 0.1% formic acid) and B (acetonitrile with 0.1% formic acid).

Mass spectrometry was performed using an Agilent 6530 UHD and Accurate-Mass Q-TOF-MS (Agilent Technologies) equipped with two electrospray ionization sources, which can operate in both positive ion mode (ESI^+^) and negative ion mode (ESI^–^). Nitrogen was used as a nebulizer gas. Le-enkephalin was used as a lock mass to ensure accuracy and reproducibility of the data. Other parameters for mass spectrometry were as follows: the scan time was 0.03 s, the inter scan time was 0.02 s, centroid data were collected ranging from 50 to 1000 m z^–1^, the source temperature was 100°C with a gas flow rate of 50 L h^–1^, the desolvation temperature was 350°C with a gas flow rate of 600 L h^–1^ (ESI^+^) or 700 L h^–1^ (ESI^–^), the capillary voltage was set to 4 KV (ESI^+^) and 3.5 KV (ESI^–^), and the sampling cone voltage was set to 35 KV (ESI^+^) and 50 KV (ESI^–^).

### Enzyme Assays

Adenosine triphosphate content (μmol g^–1^ protein^–1^) was determined by the phosphomolybdic acid colorimetric method with an ATP detection kit (Nanjing Jiancheng Bioengineering Institute, Nanjing, China). Before analysis, 0.2 *g* tissue and 1.8 ml boiling double-distilled water were put into a tube and homogenized for 3 min. Then the homogenate was boiled for 10 min at 100°C and centrifuged at 4000*g* for 10 min. The supernatant (30 μl) was collected for analysis at 636 nm using a 2100 spectrophotometer (Unico Instrument Co., Ltd., Shanghai, China).

Na^+^/K^+^-ATPase activity (U mg^–1^ protein^–1^) was determined by the inorganic phosphorus colorimetric method using a kit (Nanjing Jiancheng Bioengineering Institute). Before analysis, 0.2 *g* tissue and 1.8 ml cold normal saline were added into a tube and homogenized for 10 min. The homogenate was centrifuged at 2500*g* for 10 min at 4°C. The supernatant (50 μl) was collected for analysis at 636 nm using a 2100 spectrophotometer (Unico Instrument Co., Ltd.).

### Statistical Analysis

Normality distribution and homogeneity of variance of all data were tested by Kolmogorov–Smirnov test and Levene’s test using SPSS 19.0 software (SPSS Inc., Chicago, IL, United States). Differences in basic biological data including body weight, gonad weight, gutted body weight, gonad index, and respiratory tree weight between NBS and BS were analyzed by Student’s *t*-test. The body weight has a significant effect on physiological trait of sea cucumber. Therefore, Pearson correlation analysis was used to analyze the correlations between body weight and respiratory physiology among all individuals. We also performed analyses of covariance on respiratory physiology between NBS and BS, with the body weight as a covariate. Significant differences were considered if *P* < 0.05.

The raw metabolomic data was pretreated by Profiler (Agilent Technologies) and changed to a two-dimension matrix, which consisted of peak intensity, retention time, and molecular weight. The normalized data was analyzed using SIMCA-P v13.0 (Umetrics AB, Umea, Sweden) and SPSS v19.0 (SPSS Inc.). The orthogonal projection to latent structures discriminant analysis (OPLS-DA) and Student’s *t*-test were performed to identify the differential metabolites between NBS and BS in the respiratory tree tissue (*P* < 0.01). The biological function of metabolites was extracted from several biochemical databases including METLIN, KEGG, and HMDB.

## Results

### Gonad Development and Body Growth

**FIGURE 1:**
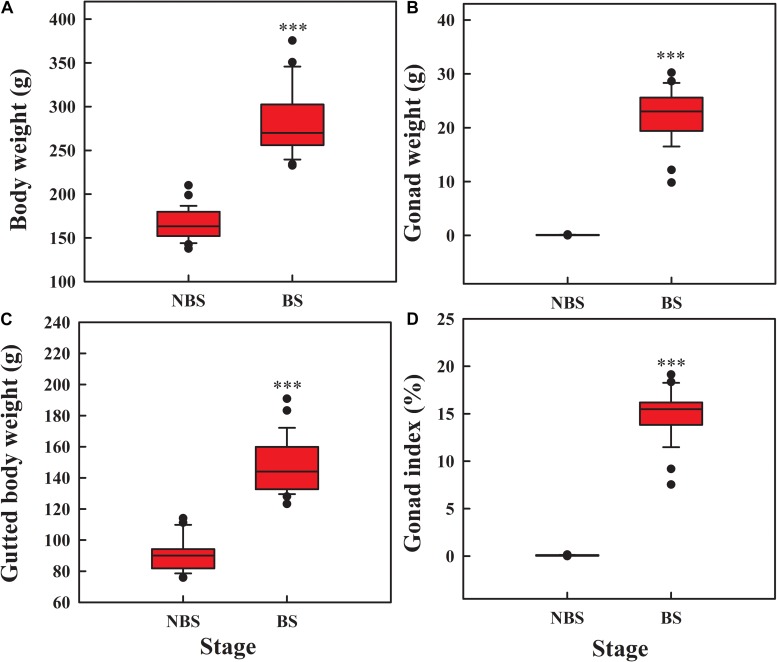
Box plot of body weight **(A)**, gonad weight **(B)**, gutted body weight **(C)**, and gonad index **(D)** of *A. japonicus* at different breeding stages. The whiskers show the range of the values, and the points show the outliers. Asterisk indicates a significant difference between the non-breeding stage (NBS) and the breeding stage (BS). ^∗∗∗^*P* < 0.001.

Compared to NBS, the body weight (Figure 1A; *t* = 15.51, *df* = 54, *P* < 0.001), gonad weight (Figure 1B; *t* = 51.876, *df* = 54, *P* < 0.001), and gutted body weight (Figure 1C; *t* = 7.26, *df* = 54, *P* < 0.001) increased significantly during reproduction. The gonad index reached to 15.06% at day 70 (Figure 1D; *t* = 27.447, *df* = 54, *P* < 0.001), suggesting the gonad is in the growth stage.

### Oxygen Consumption Rate and Respiratory Rate

**FIGURE 2:**
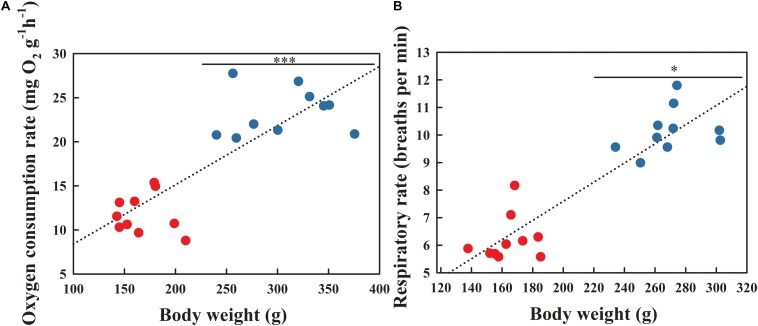
Oxygen consumption rate **(A)** and respiratory rate **(B)** of *A. japonicus* at different breeding stages. Asterisk indicates a significant difference between the non-breeding stage (NBS, red points) and the breeding stage (BS, blue points). ^∗∗∗^*P* < 0.001; ^∗^*P* < 0.05.

Pearson correlation analysis indicated that both the OCR (Figure 2A; *r* = 0.836, *P* < 0.001) and respiratory rate (Figure 2B; *r* = 0.913, *P* < 0.001) were positively correlated with body weight. The OCR during the BS was significantly higher than that in the NBS (Figure 2A; *F*_1_,_18_ = 19.583, *P* < 0.001). As expected, the respiratory rate during the BS also increased significantly compared to the NBS (Figure 2B; *F*_1_,_18_ = 5.336, *P* = 0.034).

### Organ Weight, ATP Content and Na^+^/K^+^-ATPase Enzyme Activity

**FIGURE 3:**
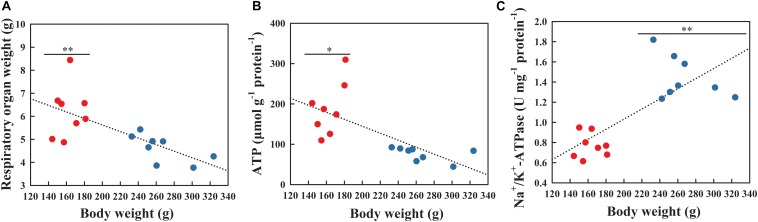
Weight **(A)**, ATP content **(B)** and Na^+^/K^+^-ATPase enzyme activity **(C)** of respiratory tree tissues in *A. japonicus* at different breeding stages. Asterisk indicates significant difference between the non-breeding stage (NBS, red points) and the breeding stage (BS, blue points). ^∗∗^*P* < 0.01; ^∗^*P* < 0.05.

Pearson correlation analysis indicated significant correlation between respiratory physiology and reproduction. The respiratory tree mass was negatively correlated with body weight (Figure 3A; *r* = −0.692, *P* = 0.003), which decreased significantly from the NBS to the BS (*t* = 3.519, *df* = 14, *P* = 0.003). The ATP content in respiratory tree tissues was also negatively correlated with body weight (Figure 3B; *r* = −0.683, *P* = 0.004), which in the NBS was significantly higher than in the BS (*F*_1_,_14_ = 5.124, *P* = 0.041). However, the Na^+^/K^+^-ATPase enzyme activity was positively correlated with body weight (Figure 3C; *r* = 0.761, *P* = 0.001), which in the BS significantly increased compared to the NBS (*F*_1_,_14_ = 20.874, *P* = 0.001).

### Identification of Metabolites

The OPLS-DA identified significant differences in the respiratory tree tissue metabolome between the BS and the NBS. The data points were separated clearly into the NBS group and the BS group in both in ESI^+^ and ESI^–^ (Supplementary Figure S1).

Combining Student’s *t*-test (*P* < 0.01), OPLS-DA model (VIP value > 1.5) and fold change (FC > 2) data, the first 10 differential metabolites were identified in ESI^+^ and ESI^–^ as key metabolites (Table 1). Eight metabolites in respiratory tree tissues increased significantly during the BS, including α-linolenic acid, 9(R)-HODE, lysoPC(16:0), lysoPC(P-16:0), lysoPC(20:0), 3-methyluridine, deoxycorticosterone (DOC) and MG(18:0e/0:0/0:0), while dehydrophytosphingosine and hypoxanthine decreased significantly. These key metabolites are involved in fatty acid metabolism, glycerophospholipid metabolism, pyrimidine metabolism, sphingolipid metabolism, steroid hormone biosynthesis and other metabolic processes. These metabolic processes are primarily associated with three physiological processes: oxidative stress, lipolysis, and hormone regulation.

**TABLE 1:** Key metabolite differences of respiratory tree tissues in *A. japonicus* between the non-breeding stage and the breeding stage.

**Metabolite**	**Ion mode**	**Mass (Da)**	**RT (min)**	**VIP value**	**FC**	***P***
***Fatty acid metabolism***						
α-Linolenic acid	ESI^+^	278.2251	13.167	1.721	2.383	0.001
9R-HODE	ESI^–^	296.2349	9.882	1.540	2.288	0.004
***Glycerophospholipid metabolism***						
LysoPC(16:0)	ESI^+^	495.3343	9.153	1.896	2.176	<0.001
LysoPC(P-16:0)	ESI^+^	479.3388	10.423	2.026	2.567	<0.001
LysoPC(20:0)	ESI^+^	551.3945	12.325	1.659	4.180	0.002
***Sphingolipid metabolism***						
Dehydrophytosphingosine	ESI^+^	315.2779	8.953	1.862	−2.505	<0.001
***Steroid hormone biosynthesis***						
Deoxycorticosterone	ESI^+^	330.2199	12.702	1.694	4.104	0.001
***Other***						
MG(18:0e/0:0/0:0)	ESI^+^	344.3295	12.757	1.655	2.248	0.002
3-Methyluridine	ESI^–^	258.0847	11.562	1.608	2.640	0.002
Hypoxanthine	ESI^–^	136.0387	1.099	1.670	−2.121	0.001

*Mass, compound molecular weight; RT, retention time in minutes; VIP value, variable importance in the projection; FC, fold change, calculated as the binary logarithm of the mean abundance of metabolites in respiratory tree samples of the breeding stage versus the non-breeding stage. LysoPC, lysophosphatidylcholine; LysoPE, lysophosphatidylethanolamine; MG, monoglyceride; 9R-HODE, 9-hydroxy-10, 12-octadecadienoic acid.*

## Discussion

As predicted, reproduction induced a significant increase in oxygen consumption in sea cucumbers. Interestingly, breeding sea cucumbers can accommodate the high oxygen demand by accelerating respiratory behavior. Therefore, the data presented here indicate that the plasticity of respiratory function is key life-history tactic to comply with the high oxygen demand during reproduction. Further, oxidative stress and hormone regulation may serve as potential mechanisms linking plasticity in respiratory function and reproduction.

### High Oxygen Demand and Respiratory Plasticity During Reproduction

The high oxygen demand in a breeding animal maybe explained by several potential hypotheses: increasing in locomotor behavior to search for mates or food (Campbell et al., 2013), maintaining growth of gonads and/or embryos (Shiku et al., 2001; Sturmey and Leese, 2003), accessory reproductive behaviors (e.g., nesting and migration) or other physiological processes needed for reproduction (Fossette et al., 2012). However, our recent studies have found that three energy-demanding activities including locomotor behavior, feeding behavior and immune function in sea cucumber were remarkably impaired over gonad development (Ru et al., 2017; Ru et al., 2018). Therefore, gonad development is a key factor resulting in increasing OCR in sea cucumber. This is because oxygen and nutrients need to be transported throughout the maternal body to support the active gonadal metabolism (Birchard et al., 1984; Quetglas et al., 2011). In addition, metabolic wastes also need to be excreted as ammonia by the maternal circulation (Birchard et al., 1984; Yang et al., 2006). Both of these processes will aggravate metabolic burden.

Plasticity in respiratory function is a key capacity of animal to copy with metabolic changes. For example, fishes can adjust their respiratory rate to elevated metabolic rate when suffering external environmental stress (Claireaux and Chabot, 2016). For internal state (e.g., reproductive condition), although many previous studies have documented elevated metabolic rates during reproduction in various species (Birchard et al., 1984; Nilsson and Råberg, 2001; Sparling et al., 2006; Costantini, 2008; Crocker et al., 2011). However, less attention has been paid on how plasticity in respiratory function may account for the high oxygen demand needed during reproduction. In our present study, we showed that high plasticity of breathing frequency is an effective tactic to adjust oxygen intake to needs in sea cucumber. Similar to previous studies focusing on external factors (Xie et al., 2017), our study also support the hypothesis that high metabolic rate triggers the change of breathing function, and this is only possible by having the capacity to flexibly adjust respiratory frequency.

Variations of ATP content maybe key to better understand the relationship between plasticity in respiratory function and increasing oxygen demand (Finkel, 2006). As the respiratory rate increased during the BS, there was a reduction in ATP content in the respiratory organs. This observation suggests that the accelerating breathing behavior needs large amounts of ATP to sustain it. But the increased activity of Na^+^/K^+^-ATPase and decreased organ mass suggest that the gravid sea cucumber changes its energy utilization tactic and mitochondrial metabolism to produce more ATP to serve the increased ATP consumption. More specifically, the increased metabolite levels of monoglyceride (MG) and fatty acid indicate that the stored lipid resource in the respiratory organ is being consumed (Zimmermann et al., 2009). This implies that energy allocation to the respiratory organ is decreased or even ceased (Ji et al., 2008; Zhao et al., 2014). In other words, gravid sea cucumbers may consume both stored fat and direct food intake as the energy source to support respiratory behavior during reproduction. This changing energy utilization tactic is an adaptive compromise that balances the energy trade-off between reproduction and other physiological functions (Harshman and Zera, 2007; Ru et al., 2017).

Reproduction brings inevitable physical and physiological burdens to the animal, and may even impair survival (Harshman and Zera, 2007; Speakman, 2008). The results presented in this report showed that sea cucumbers can adjust their respiratory physiology to respond to reproductive challenges. Similar results were also reported in other species. For example, turtles (*Caretta caretta*) change locomotory function to meet thermal demand (Fossette et al., 2012). Similarly, changing digestive function in Koala (*Phascolarctos cinereus*) was reported to indicate a response in the animal to nutrient demand (Krockenberger and Hume, 2007). Thus, we suggest that plasticity of respiratory function is an important life-history tactic to accommodate for variation in oxygen demand during reproduction in sea cucumbers.

### Potential Mechanisms Linking Reproduction and Respiratory Plasticity

In spite of complex physiological mechanisms, oxidative stress may play an important role underlying plasticity of respiratory function. LysoPC is a key metabolic marker of oxidative stress (Ha et al., 2012; Kim et al., 2013). We observed that three lysoPCs increased in sea cucumbers during the breeding season, implying that oxidative damage has occurred in the respiratory organ. Oxidative stress is caused by physiological by-products (i.e., reactive oxygen species) of mitochondrial metabolism, and oxidative stress occurs at high metabolic status when reactive oxygen species is overproduction (Speakman, 2008). Interestingly, oxidative stress has been widely considered as a physiological cost of reproduction (Alonso-Alvarez et al., 2004; Costantini, 2008; Xu et al., 2014; Sharick et al., 2015), and recent studies even found that oxidative stress differs significantly between organs during reproduction (Yang et al., 2013; Xu et al., 2014). However, the direct evidence linking reproduction, organ function, and oxidative stress is still poorly understood (Speakman et al., 2015). In our present study, we suggest that the oxidative damage in respiratory organ is a physiological consequence of high metabolic status in sea cucumber, because breeding sea cucumber need produce ATP to sustain high respiratory rate during reproduction.

Likewise, elevated DOC may another result of oxidative stress and changing metabolic states during reproduction. DOC and its derivatives are key mineralocorticoid hormones associated with osmoregulation, energy metabolism and immune function in teleost fish. Unexpectedly, we detected the existence of DOC in a marine invertebrate using untargeted metabolomics approach. More surprisingly, we observed that DOC level in respiratory tree significant increased during reproduction. There are two reasonable explanations for the elevated hormone level in our study. On the one hand, DOC boosts Na^+^/K^+^-ATPase activity in order to produce sufficient ATP to sustain the accelerated respiratory behavior (Kiilerich et al., 2011). On the other hand, DOC is an endocrine regulator to maintain the immune balance during reproduction (Mathieu et al., 2013; Milla et al., 2018). For example, DOC can minimize the negative consequence of the occurring oxidative stress. Therefore, consistent with previous studies focusing on corticosterone (Michael Romero, 2002; Love et al., 2004), a metabolic derivative of DOC, we also suggested that DOC is an endocrine mediator for sea cucumbers to maximize their fitness during their life-span. Because our result indicated that DOC would play a key role in balancing the life-history trade-off between current reproductive success (i.e., maintain the high metabolic rate) and parental survival (i.e., maintain the immune balance).

In addition, the functions of metabolites including dehydrophytosphingosine and hypoxanthine in breeding animals are still unknown. However, the two metabolites may play key roles in maintaining the growth, survive, division and signal transduction of cells (Hannun and Obeid, 2008; Obajimi and Melera, 2008; Chen et al., 2012). Therefore, the observed interaction patterns between reproduction, physiological plasticity and cell function still require future study.

## Conclusion

In conclusion, the data show that the plasticity of respiratory function is an important tactic to satisfy the increasing oxygen consumption in breeding sea cucumbers. Oxidative stress and hormone regulation are potential physiological mechanisms underlying respiratory plasticity during reproduction. Therefore, this study provides new insight into how animals change their respiratory physiology to meet costly reproductive demands.

## Data Availability Statement

All datasets generated for this study are included in the article/Supplementary Material.

## Ethics Statement

All procedures involved in animal collection, rearing and dissection were performed under the Guideline of Ethical Regulations of Animal Welfare of the Institute of Oceanology, Chinese Academy of Sciences (IOCAS 2013.3). Our research protocols were approved by the Animal Welfare Committee of the IOCAS.

## Author Contributions

XR performed the research and wrote the manuscript. SL provided cameras and sea cucumbers. LZ and HY supervised this study.

## Conflict of Interest

The authors declare that the research was conducted in the absence of any commercial or financial relationships that could be construed as a potential conflict of interest.

## Supplementary Material

FIGURE S1 The orthogonal projection to latent structures discriminant analysis (OPLS-DA) scores plot of respiratory tree metabolites from the non-breeding stage (blue squares) and the breeding stage (yellow triangles) in positive ion mode **(A)** and negative ion mode **(B)**.
