# Supplementary figures and images for: Plasticity of Respiratory Function Accommodates High Oxygen Demand in Breeding Sea Cucumbers
Source: Front Physiol. 2020 Apr 2;11:283. doi: 10.3389/fphys.2020.00283 (PMC7145410; doi:10.3389/fphys.2020.00283)

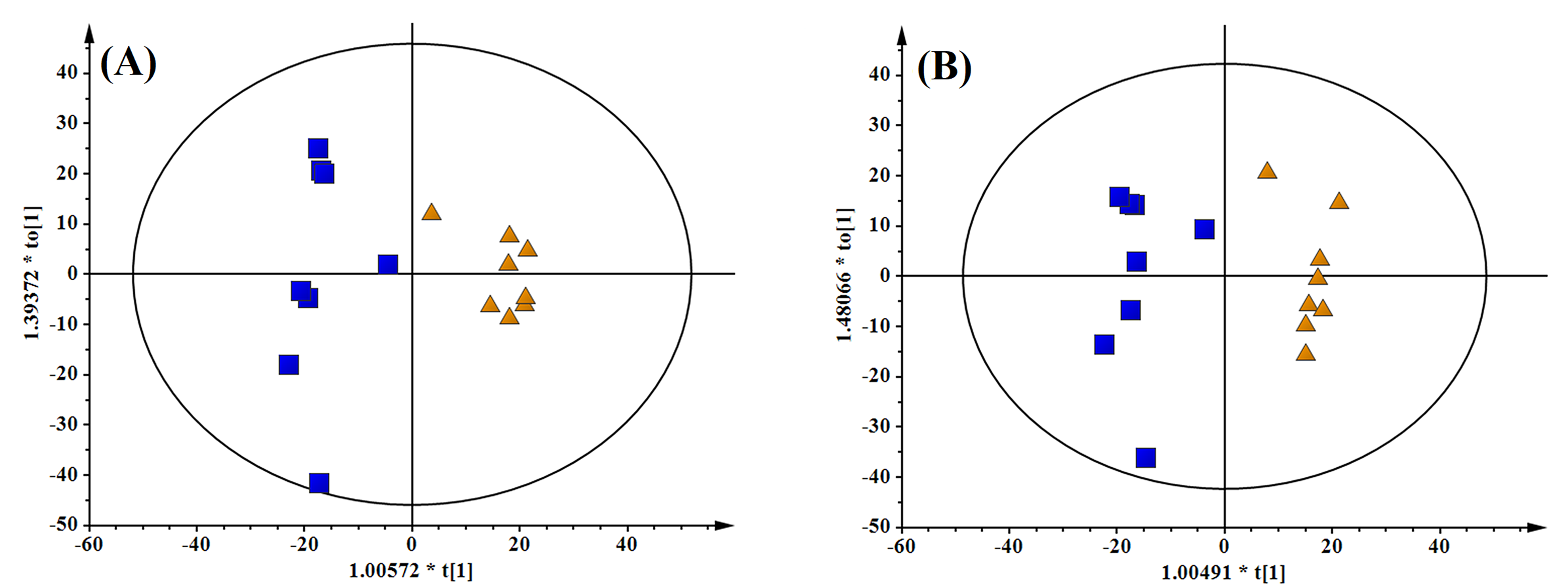

Supplement: FIGURE S1 — The orthogonal projection to latent structures discriminant analysis (OPLS-DA) scores plot of respiratory tree metabolites from the non-breeding stage (blue squares) and the breeding stage (yellow triangles) in positive ion mode (A) and negative ion mode (B). [file Image_1.TIF]
